# Improving the Human Hazard Characterization of Chemicals: A Tox21 Update

**DOI:** 10.1289/ehp.1205784

**Published:** 2013-04-19

**Authors:** Raymond R. Tice, Christopher P. Austin, Robert J. Kavlock, John R. Bucher

**Affiliations:** 1 Division of the National Toxicology Program, National Institute of Environmental Health Sciences, National Institutes of Health, Department of Health and Human Services, Research Triangle Park, North Carolina, USA; 2 National Center for Advancing Translational Sciences, National Institutes of Health, Department of Health and Human Services, Bethesda, Maryland, USA; 3 National Center for Computational Toxicology, Office of Research and Development, U.S. Environmental Protection Agency, Research Triangle Park, North Carolina, USA

**Keywords:** chemical hazard characterization, computational biology, high throughput testing, *in vitro* models, systems biology, Tox21

## Abstract

Background: In 2008, the National Institute of Environmental Health Sciences/National Toxicology Program, the U.S. Environmental Protection Agency’s National Center for Computational Toxicology, and the National Human Genome Research Institute/National Institutes of Health Chemical Genomics Center entered into an agreement on “high throughput screening, toxicity pathway profiling, and biological interpretation of findings.” In 2010, the U.S. Food and Drug Administration (FDA) joined the collaboration, known informally as Tox21.

Objectives: The Tox21 partners agreed to develop a vision and devise an implementation strategy to shift the assessment of chemical hazards away from traditional experimental animal toxicology studies to one based on target-specific, mechanism-based, biological observations largely obtained using *in vitro* assays.

Discussion: Here we outline the efforts of the Tox21 partners up to the time the FDA joined the collaboration, describe the approaches taken to develop the science and technologies that are currently being used, assess the current status, and identify problems that could impede further progress as well as suggest approaches to address those problems.

Conclusion: Tox21 faces some very difficult issues. However, we are making progress in integrating data from diverse technologies and end points into what is effectively a systems-biology approach to toxicology. This can be accomplished only when comprehensive knowledge is obtained with broad coverage of chemical and biological/toxicological space. The efforts thus far reflect the initial stage of an exceedingly complicated program, one that will likely take decades to fully achieve its goals. However, even at this stage, the information obtained has attracted the attention of the international scientific community, and we believe these efforts foretell the future of toxicology.

Thousands of chemicals to which humans are exposed have inadequate data on which to predict their potential for toxicological effects. However, dramatic technological advances in molecular and systems biology, computational toxicology, and bioinformatics have provided researchers and regulators with powerful new public health tools [National Research Council (NRC) 2006, 2007]. High content screening (HCS) and high throughput screening (HTS) techniques are now routinely used in conjunction with computational methods and information technology to probe how chemicals interact with biological systems, both *in vitro* and *in vivo*. Progress is being made in recognizing the patterns of response in genes and pathways induced by certain chemicals or chemical classes that might be predictive of adverse health outcomes in humans. However, as with any new technology, both the reliability and the relevance of the approach need to be demonstrated in the context of current knowledge and practice.

In 2008, in response to the National Academy of Sciences’ (NAS) report *Toxicity Testing in the 21st Century, a Vision and a Strategy* (NRC 2007), Collins et al. (2008) outlined a collaboration between the National Institute of Environmental Health Sciences (NIEHS)/National Toxicology Program (NTP), the U.S. Environmental Protection Agency’s (EPA) National Center for Computational Toxicology (NCCT), and the National Human Genome Research Institute (NHGRI)/National Institutes of Health (NIH) Chemical Genomics Center (NCGC) (now located within the National Center for Advancing Translational Sciences) to develop a vision and devise an implementation strategy to shift the assessment of chemical hazards from traditional experimental animal toxicology studies to target-specific, mechanism-based, biological observations largely obtained using *in vitro* assays. In mid-2010, the U.S. Food and Drug Administration (FDA) joined the collaboration, which is known informally as Tox21.

The Tox21 partner agencies (Collins et al. 2008) agree to collaborate to

Research, develop, validate, and translate innovative compound testing methods to characterize toxicity pathways.
Identify compounds, assays, informatic analyses, and targeted testing needed to support development of the new methods.
Identify patterns of compound-induced biological response in order to characterize toxicity pathways, facilitate cross-species extrapolation, and model low-dose extrapolation.
Prioritize compounds for more extensive toxicological evaluation.
Develop predictive models for biological response in humans.
Make all data publicly available.

The purpose of this review is to outline the efforts of the U.S. EPA, the NCGC, and the NTP up to the time the FDA joined the collaboration; to describe the approaches taken to develop the science and technologies currently being used; to assess the current status; and to identify problems that could impede further progress as well as approaches to address those problems.

To support the goals of Tox21, four working groups—Compound Selection, Assays and Pathways, Informatics, and Targeted Testing—were established; a representative of each Tox21 partner serves as a co-chair on each working group. The working groups reflect the different components of the NAS vision (NRC 2007) and cooperatively address the four major focus areas necessary to bring about this paradigm shift [see NIEHS (2012) for additional information on the approaches and components of Tox21].

## Discussion

*Chemical selection and lessons learned*. Developing a comprehensive set of substances (i.e., a compound library) of toxicologic concern is critical to the ultimate ability of Tox21 to develop relevant prioritization schemes and prediction models. Ideally, any library should be populated with substances of known identity and purity that are compatible with the solvent of choice for the assay platforms being used. In 2006, the NTP and the U.S. EPA established at the NCGC “proof of principle” libraries of 1,408 and 1,462 compounds, respectively, with each compound dissolved and stored in dimethyl sulfoxide (DMSO), to be evaluated for activity in 1,536-well plate quantitative high throughput screens (qHTS), as described by Inglese et al. (2006). In traditional HTS, compounds are tested at a single concentration and the results are therefore burdened by frequent false negatives or positives. In contrast, in qHTS, many thousands of compounds are screened in a single experiment across a broad concentration range in order to generate concentration–response curves. The method identifies compounds with a wide range of activities with a much lower false-positive or false-negative rate. The resulting concentration–response curves can be classified to rapidly identify actives and inactives with a variety of potencies and efficacies, producing rich data sets that can be mined for reliable biological activities.

The identities and structures of the compounds in these libraries are available in PubChem (http://pubchem.ncbi.nlm.nih.gov/) using the assay identification number (AID). To evaluate within-assay reproducibility, each library included a number of compounds in duplicate (based on structure: 66 for the NTP; 77 for the U.S. EPA) although the two libraries contained 411 compounds in common, generally representing different suppliers and/or lots when from the same supplier. During examination of the data generated using these libraries, issues were identified (e.g., inaccurate information on certificates of analysis accompanying purchased chemicals, lack of compound stability under the conditions of storage and use) that informed efforts during the development of the much larger compound library to be screened as part of Tox21 Phase II using a recently established, dedicated Tox21 robotics facility at the NCGC (NIEHS 2011a).

**Table 1:** qHTS assays used at the NCGC during Tox21 Phase I.

Assay type or acronym	Assay description	Library screened	PubChem AID	Reference^*a*^
Phenotypic cell-based assay readouts
Caspase 3/7	Induction of caspase 3 and 7 in 13 cell types	NTP	654–661, 663–667	Huang etal. 2008
Caspase 3/7	Induction of caspase 3 and 7 in 81 human lymphoblastoid cell lines	NTP	588813	Lock etal. 2012
Cytotoxicity	Levels of ATP in 13 cell types	NTP	421, 426, 427, 433–435, 540–546	Huang etal. 2008; Xia etal. 2008
Cytotoxicity	Levels of ATP in 81 human lymphoblastoid cell lines from trios (parents and child)	NTP	588812	Lock etal. 2012
Cytotoxicity	Levels of ATP in 26 human lymphoblastoid cell lines produced from twins	NTP	921, 965–989	
Mitochondrial toxicity	Mitochondrial membrane potential	NTP	651754, 651755	Sakamuru etal. 2012
Cell signaling assays
Anthrax lethal factor	Assay for anthrax lethal toxin internalization	NTP^*b*^	912	
AP1	Induction of activator protein 1	NTP^*b*^	357	
ARE	Antioxidant response element	NTP	651741	Shukla etal. 2012
CRE	Cyclic AMP response element used to measure forskolin-induced signaling	NTP^*b*^	662	
HIF	Assay for identification of inducers of hypoxia inducible factor 1	NTP^*b*^	2120	Xia etal. 2011
HRE	Assay for identification of antagonists for hypoxia response element signaling pathway	NTP^*b*^	915	
LDR	Assay (Locus DeRepression) for epigenetic modulators	NTP^*b*^	597	
NPS	Assay for antagonists of the neuropeptide S receptor: cAMP signal transduction	NTP^*b*^	1461	
Proteasome	Assay for inhibitors of the ubiquitin-proteasome pathway	NTP^*b*^	526	
SMN2	Assay for enhancers of SMN2 (survival motor neuron protein) splice variant expression	NTP^*b*^	1458	
Thalassemic	Assay for modulators of hemoglobin β chain splicing	NTP^*b*^	925	
TSH	Assay for agonists and antagonists of the thyroid stimulating hormone receptor	NTP^*b*^	926, 938, 504813	
DNA damage cell-based assays
ATAD5	DNA damage response element	NTP^*b*^	493106, 504467	Fox etal. 2013
Chicken DT40 isogenic DNA repair deficient cell lines	Differential cytotoxicity (measured as levels of ATP) in seven chicken DT40 isogenic DNA repair deficient cell lines vs. the parental cell line	NTP, U.S. EPA	651838	Part published by Yamamoto etal. 2011
p53	DNA damage and other stress response element	NTP	651743	
Immune response cell-based assays
IL-8	Assay to measure induction of interleukin 8	NTP, U.S. EPA	651758	
TNFα	Assay to measure induction of tumor necrosis factor-α	NTP, U.S. EPA	651757	
Other cell-based assays
ERK	Assay for inhibitors of the extracellular signal-regulated kinase (ERK) signaling pathway using a homogeneous screening assay	NTP^*b*^	995	
JNK3	Assay for the modulation of c-Jun N-terminal kinase 3	NTP^*b*^	530	
NFκB	Assay for the induction of nuclear factor-κB	NTP, U.S. EPA	651749	
Drug metabolism biochemical assays
CYP1A2	Assay for inhibitors and substrates of cytochrome P450 1A2	NTP, U.S. EPA	410	Sun etal. 2012
CYP2C19	Assay for inhibitors and substrates of cytochrome P450 2C19	NTP, U.S. EPA	899	Sun etal. 2012
CYP2C9	Assay for inhibitors and substrates of cytochrome P450 2C9	NTP, U.S. EPA	883	Sun etal. 2012
CYP2D6	Assay for inhibitors and substrates of cytochrome P450 2D6	NTP, U.S. EPA	891	Sun etal. 2012
CYP3A4	Assay for inhibitors and substrates of cytochrome P450 3A4	NTP, U.S. EPA	884	Sun etal. 2012
Nuclear receptor cell-based assays
hAR	Assay for human androgen receptor (partial) agonists and antagonists	NTP, U.S. EPA	588515, 588516	Huang etal. 2011a
hAhR	Assay for human aryl hydrocarbon receptor (partial) agonists and antagonists	NTP, U.S. EPA	651777	
hERα	Assay for human estrogen α receptor (partial) agonists and antagonists	NTP, U.S. EPA	588513, 588514	Huang etal. 2011a
hFXR	Assay for human farnesoid X receptor (partial) agonists and antagonists	NTP, U.S. EPA	588526, 588527	Huang etal. 2011a
hGR	Assay for human glucocorticoid receptor (partial) agonists and antagonists	NTP, U.S. EPA	588532, 588533	Huang etal. 2011a
hPPAR-α	Assay for human peroxisome proliferator-activated receptor α (partial) agonists and antagonists	NTP, U.S. EPA	651778	
hPPAR-δ	Assay for human peroxisome proliferator-activated receptor δ (partial) agonists and antagonists	NTP, U.S. EPA	588534, 588535	Huang etal. 2011a
hPPAR-γ	Assay for human peroxisome proliferator-activated receptor γ (partial) agonists and antagonists	NTP, U.S. EPA	588536, 588537	Huang etal. 2011a
rPXR	Assay for rat pregnane X receptor (partial) agonists and antagonists	NTP, U.S. EPA	651751	
hRORγ	Assay for human retinoic acid-related orphan receptor γ (partial) agonists and antagonists	NTP	651802	
mRORγ	Assay for mouse retinoic acid-related orphan receptor γ antagonists	NTP^*b*^	2546, 2551	
hRXR	Assay for human retinoid X receptor (partial) agonists and antagonists	NTP, U.S. EPA	588544, 588546	Huang etal. 2011a
hTRβ	Assay for human thyroid hormone receptor (partial) agonists and antagonists	NTP, U.S. EPA	588545, 588547	Huang etal. 2011a
VDR	Assay for human vitamin D receptor (partial) agonists and antagonists	NTP, U.S. EPA	588541, 588543	Huang etal. 2011a
Isolated molecular targets
12hLO	Assay for inhibitors of 12-human lipoxygenase	NTP^*b*^	1452	
15hLO2	Assay for inhibitors of 15-human lipoxygenase 2	NTP^*b*^	881	
ALDH1A1	Assay for inhibitors of aldehyde dehydrogenase 1 family, member A1	NTP^*b*^	1030	
α-glucosidase	Assay for inhibitors of human α-glucosidase as a potential chaperone treatment of Pompe Disease	NTP^*b*^	1466	
α-galactosidase (human tissue)	Assay for inhibitors of human α-galactosidase at pH 4.5	NTP^*b*^	1467	
AMPC	Promiscuous and specific inhibitors of AmpC β-lactamase (assay with and without detergent)	NTP^*b*^	584, 585	
APE1	Assay for inhibitors of the human apurinic/apyrimidinic endonuclease 1	NTP^*b*^	1705	
BRCA	Assay for inhibitors of *BRCT*-phosphoprotein interaction (green fluorophore, red fluorophore)	NTP^*b*^	875, 892	
Caspase-1 enzyme	Assay for allosteric/competitive inhibitors of caspase-1	NTP^*b*^	900	
Caspase-7 enzyme	Assay for allosteric/competitive inhibitors of caspase-7	NTP^*b*^	889	
CBFβ-RUNX1	Assay for compounds blocking the interaction between* CBF-*β and *RUNX1* for the treatment of acute myeloid leukemia	NTP^*b*^	1477	
Cruzain	Assay for promiscuous and specific inhibitors of cruzain (with and without detergent)	NTP^*b*^	1476, 1478	
DNA polymerase III	Assay for inhibitors of DNA polymerase III holoenzyme system	NTP^*b*^	603	
Glucocerebrosidase	Assay for inhibitors that could potentially act as molecular chaperones on mutant forms	NTP^*b*^	360	
HADH560	Assay for Inhibitors of hydroxyacyl-coenzyme A dehydrogenase, type II	NTP^*b*^	886	
hERG	Assay for inhibitors of human ether-a-go-go-related gene potassium (hERG) channel activity	NTP^*b*^	588834	Xia etal. 2011
HPGD	Assay for inhibitors of 15-hydroxyprostaglandin dehydrogenase	NTP^*b*^	894	
HSDB130	Assay for inhibitors of hydroxysteroid (17-β) dehydrogenase 4	NTP^*b*^	893	
HSP90	Assay for disrupters of an heat shock protein 90kDa α (cytosolic) family co-chaperone interaction	NTP^*b*^	595	
Huntington	Assay to Identify dual action probes in a cell model of Huntington: cytoprotection (ATP)	NTP^*b*^	1471	
IMPase	Assay for identifying the cell-membrane permeable inositol monophosphatase inhibitors	NTP^*b*^	901	
i-RGS	Assay for inhibitors of G-protein signaling protein RGS12 GoLoco motif activity (red fluorophore)	NTP^*b*^	880	
*O*-Glc NAc transferase	Assay for inhibitors of *O*-Glc *N*-acetylcysteine transferase (sOGT)	NTP^*b*^	447	
p53 synthetic lethal	Screen for compounds that selectively target cancer cells with p53 mutations by measuring cytotoxicity of p53ts cells at the nonpermissive (32°C) and permissive (39°C) temperatures	NTP^*b*^	902, 924	
PK	Assay for inhibitors of *Leishmania mexicana* pyruvate kinase (LmPK)	NTP^*b*^	1721	
PRX	Assay for inhibitors of *Schistosoma* *mansoni* peroxiredoxins (Prx2)	NTP^*b*^	448	
RECQ1 helicase	Assay for inhibitors of the human RecQ-Like DNA helicase 1 (RECQ1)	NTP^*b*^	2549	
SMN2	Assay for enhancers of SMN2 (survival motor neuron protein) splice variant expression	NTP^*b*^	1458	
Tau	Assay for Tau filament binding	NTP^*b*^	596	
TDP1	Assay for inhibitors of tyrosyl-DNA phosphodiesterase	NTP^*b*^	485290	
TR (protein antagonist)	Assay for inhibitors of the interaction of thyroid hormone receptor and steroid receptor coregulator 2	NTP^*b*^	1469	
YjeE:ADP binding	Assay for inhibitors of YjeE, essential *Escherichia coli* protein of unknown function that binds ADP	NTP^*b*^	605	
^***a***^No entry indicates that the data have not yet been used in a publication by the Tox21 partners. ^***b***^NTP compounds were included in a much larger library screened by the NCGC as part of the Molecular Libraries Initiative.				

The Tox21 Phase II compound library includes structurally defined compounds intended to broadly capture chemical and toxicological “space.” The libraries include compounds with extensive to no toxicological information and with use, production, chemical class identity, and/or environmental exposure patterns that make them of potential concern to regulatory agencies. To produce this compound library, an initial list of approximately 120,000 compounds was culled to approximately 11,000 unique compounds with known structures. The physical property cutoffs for the Phase II library were a molecular weight range of 100–1,000, a vapor pressure of < 10 Pa, and a calculated log *p*-value of –2 to 6. The desired solubility in DMSO was 20 mM, but some compounds of special interest, soluble only at a lower concentration, have been included. The library contains the compounds in the U.S. EPA’s ToxCast™ Phases I and II, including approximately 150 pharmaceuticals that failed in clinical trials (U.S. EPA 2013f). These failed pharmaceuticals were provided to the U.S. EPA by Pfizer (New York, NY), Merck (Summit, NJ), GlaxoSmithKline (Research Triangle Park, NC), Sanofi (Bridgewater, NJ), Roche (Indianapolis, IN), and Astella (Northbrook, IL), with the understanding that the identity, structure, and toxicity data would be made public. The NCGC contribution to the library is the recently developed NCGC Pharmaceutical Collection (NPC), a comprehensive, publicly accessible collection of approved and investigational drugs for HTS (Huang et al. 2011b). The NPC contains approximately 3,500 small molecules that have been approved for clinical use by the United States (FDA), European Union (European Medicines Agency), Japan (Minister of Health, Labor, and Welfare), and Canada (Health Canada) and that are amenable to HTS screening.

The completion of the Tox21 Phase II library was announced in December 2011 (NIEHS 2011b). This library contains > 10,000 (10K) compounds [8,193 of which are unique; see U.S. EPA (2013e) for the complete list]; the compounds fall into classes that include, among others, industrial chemicals, sunscreen additives, flame retardants, pesticides and selected metabolites, plasticizers, solvents, food additives, natural product components, drinking water disinfection by-products, preservatives, therapeutic agents, and chemical synthesis by-products. Although the focus of the 10K library is on individual compounds with known structures, a few hundred formulations prepared from sets of 8–62 compounds with selection based on estrogen receptor activity, androgen receptor activity, and *in vitro* cytotoxicity profiles have been included, as well as each individual constituent, to explore how a mixtures library could be established and the resulting HTS data evaluated as part of Tox21. Another future plan is to establish a library with water as a solvent for hydrophilic compounds that are relatively insoluble in DMSO.

To evaluate within-run reproducibility, a set of 88 broadly bioactive compounds is included in duplicate on each 1,536-well assay plate. The library also includes multiple samples of many compounds, providing another measure of compound and assay variability. The 10K library is being screened three times in each qHTS assay at the NCGC, with compounds in a different well location during each run, to better evaluate assay reliability and to increase the ability to distinguish between weak active and inactive compounds.

To address compound identity and purity, to confirm the stock solution concentration (generally 20 mM), and to determine compound stability in DMSO under the storage conditions used, quality control analysis of the entire library is being conducted using a tiered approach. First, a high throughput, HPLC system with multiple detectors [mass spectrometry, ultraviolet diode array, evaporative light scattering detection (ELSD), and chemiluminescent nitrogen detection (CLND)] is being used for identity characterization and purity estimation. Identity confirmation is performed by a matching molecular ion in the mass spectrum with the desired compound; purity analysis is conducted with the ELSD. For compounds containing nitrogen, the CLND provides quantitation of the compound concentration. This system does not work well for some of the more volatile compounds or for those that will not properly ionize in the mass spectrometer. As needed, follow-up analyses are being conducted by gas chromatography with mass spectrometry and other analytical techniques. The stability of the compounds under the conditions of use will be determined also. Chromatographic and quality control data for all components of the Tox21 Library will be linked to the master chemical list and the qHTS data and made publicly available.

**Figure 1:**
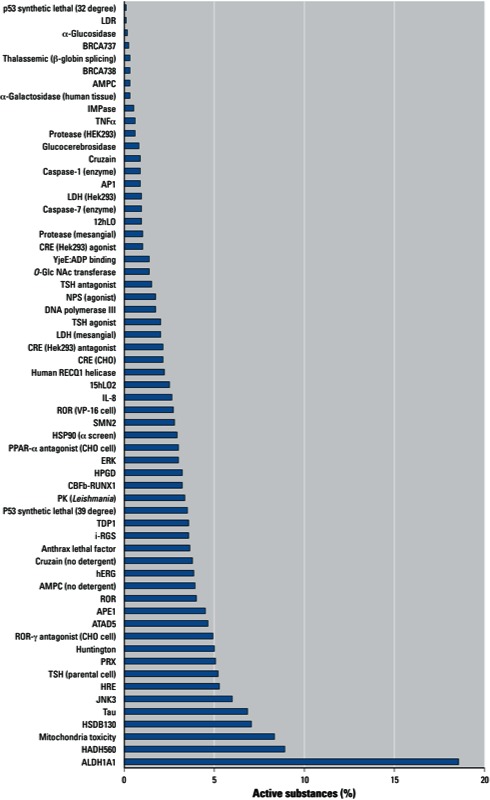
Results of phenotypic assays of the NTP 1,408-compound library showing the percentage of substances classified as active in each assay. Abbreviations: 12hLO, 12-human lipoxygenase; ALDH1A1, aldehyde dehydrogenase 1 family, member A1; AMPC, b-lactamase/d-alanine carboxypeptidase; AP1, activator protein 1; APE1, apurinic/apyrimidinic endonuclease 1; ATAD5, DNA damage response element; BRCA, breast cancer, early onset; CHO, Chinese hamster ovary; CRE, cAMP response element; ERK, extracellular signal-regulated kinase; HADH560, hydroxyacyl-coenzyme A dehydrogenase, type II; HEK, human embryonic kidney; hERG, human ether-a-go-go–related gene potassium channel; HPGD, hydroxyprostaglandin dehydrogenase; HRE, hypoxia response element; HSDB, hydroxysteroid (17-b) dehydrogenase; HSP90, heat shock protein 90kDa a; IL-8, interleukin 8; IMPase, inositol monophosphatase; i-RGS, G-protein signaling protein RGS12; JNK, c-Jun N-terminal kinase; LDH, l-lactate dehydrogenase; LDR, Locus DeRepression; NPS, neuropeptide S; O-Glc NAc transferase, O-Glc NAc *N*-acetylcysteine transferase (sOGT); PK, pyruvate kinase; PPAR-a, peroxisome proliferator-activated receptor a; ROR, retinoic acid-related orphan receptor; PRX, peroxiredoxins; SMN2, survival motor neuron protein; TDP1, tyrosyl-DNA phosphodiesterase; TNFa, tumor necrosis factor-a; TSH, thyroid stimulating hormone; YjeE:ADP binding, essential *Escherichia coli* protein of unknown function that binds adenosine diphosphate.

Quantitation of the compounds in DMSO is complex given the diversity of their chemical properties. To date, we have not identified a cost-effective approach for confirming the concentration of each compound under test conditions in the 1,536-well format. Accomplishing this requires additional sensitivity because the plates are assayed at far lower concentrations than the “source” plate, and the analytical system has to be compatible with water and buffers.

*Assay selection and lessons learned*. At the NCGC, using qHTS, tens of thousands of compounds can be rapidly screened at multiple concentrations (typically 15 concentrations, from ~ 0.5 nM to ~ 92 μM) to yield concentration–response curves defining compound activity. Assay selection during Phase I was constrained by the availability of suitable assays, both from a technological and a biological perspective. Essentially, Phase I screening at the NCGC was a pilot study to evaluate assay performance, methods of assay protocol optimization, and the extent to which protocols could be varied without compromising results. The qHTS data generated were also used to develop appropriate statistical analysis procedures to allow automated evaluation of thousands of qHTS concentration curves to identify actives and inactives in different kinds of assays. In addition, a number of strategies for orthogonal (i.e., the same biological outcome on a different assay platform) or follow-up screens to confirm and extend the results obtained were explored (Xia et al. 2009, 2011).

Assay selection was accomplished via several mechanisms. Initially, four commercially available cell-based assays were selected to evaluate the suitability of the qHTS approach in the 1,536-well format for screening a non-drug–like compound library. These assays were the Promega CellTiter Glo® cell viability assay, which measures intracellular ATP levels, and the Promega Caspase Glo® 3/7, 8, and 9 assays, which measure apoptosis (Promega Corporation, Madison, WI). The CellTiter Glo® cell viability assay was used first by screening the NTP 1,408-compound library for cytotoxicity in 13 cell types (9 human, 2 rat, 2 mouse) (Xia et al. 2008). The cell types originated from different tissues and included cell lines, cell strains, and primary cell populations. As anticipated, over the concentration range tested, there were compounds that were cytotoxic in all cell types. However, there were also compounds that were uniquely cytotoxic to only one or a few cell types. Similar results were obtained when the NTP 1,408 compounds were screened for apoptosis in the same cell types (Huang et al. 2008). These results indicated that no single cell type would be universally informative for cytotoxicity or apoptosis but that the use of multiple cell types would allow compounds to be binned by their pattern of response (Huang et al. 2008; Xia et al. 2008).

**Figure 2:**
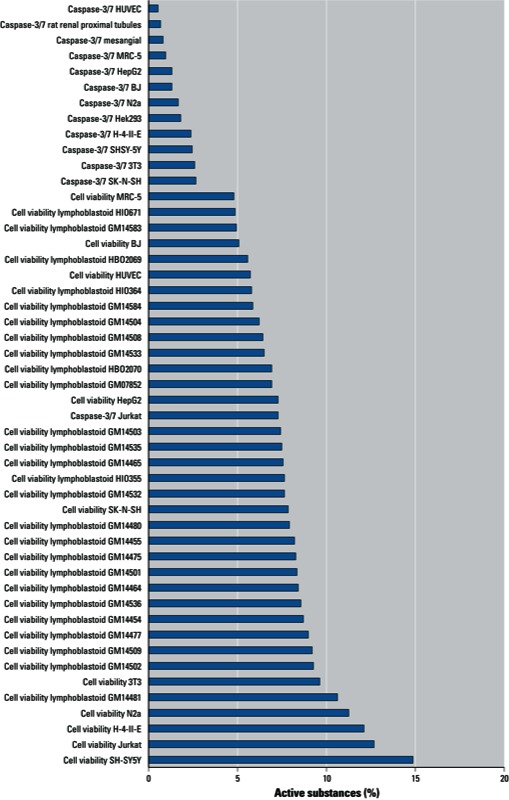
Results of pathway target assays of the NTP 1,408-compound library showing the percentage of substances classified as active in each assay.

**Figure 3:**
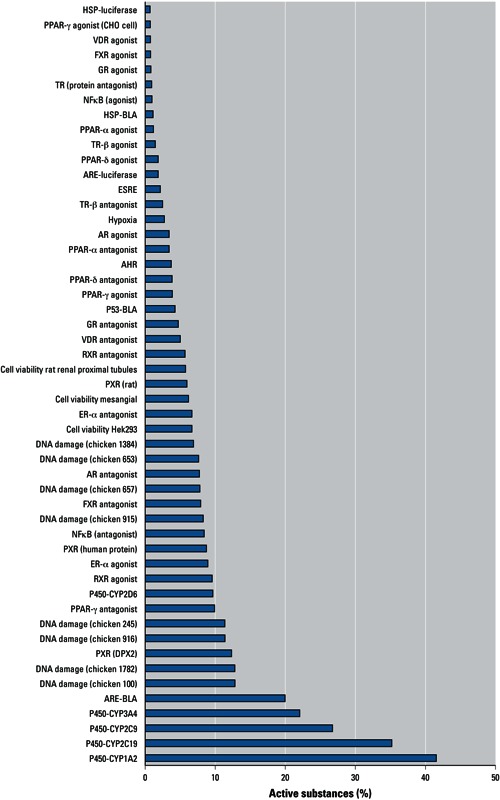
Results of nuclear receptor, DNA damage, cytochrome-p450, and miscellaneous assays of the NTP 1,408- and the U.S. EPA 1,462-compound libraries showing the percentage of substances classified as active in each assay. Abbreviations: AHR, aryl hydrocarbon receptor; AR, androgen receptor; ARE, antioxidant response element; ER, estrogen receptor; esrE, *Escherichia coli* strain K-12 substrain MG1655; FXR, farnesoid X receptor; GR, glucocorticoid receptor; HSP, heat shock protein; NFkB, nuclear factor-kB; PPAR, peroxisome proliferator-activated receptor; PXR, pregnane X receptor; RXR, retinoid X receptor; TR, thyroid hormone receptor; VDR, vitamin D receptor.

Upon completion of these assays, additional assays were added to the screening effort. In addition to assays selected by the Tox21 partners, the NTP compound library was screened in assays conducted at the NCGC as part of the Molecular Libraries Screening Initiative (NIH 2013a). The qHTS assays in which the NTP and/or U.S. EPA libraries were screened during Phase I are listed in Table 1. Figures 1–3 show the percentage of compounds classified as active in each of the phenotypic assays (NTP 1,408-compound library); the pathway/target assays (NTP 1,408-compound library); and the nuclear receptor, DNA damage, cytochrome-p450, and miscellaneous assays (NTP 1,408- and EPA 1,462-compound libraries). The substances were screened at 15 concentrations and classified as active, inconclusive, or negative, as described by Xia et al. (2011). Briefly, concentration–response titration points for each compound were fitted to a four-parameter Hill equation (Hill 1910), yielding half maximal concentration (AC_50_) and maximal response (efficacy) values. The compounds were designated as class 1–4 according to the type of concentration–response curve observed (Inglese et al. 2006). Curve classes are heuristic measures of data confidence, classifying concentration–responses on the basis of efficacy, the number of data points observed above background activity, and the quality of fit. To facilitate analysis, each curve class was combined with an efficacy cutoff and converted to a numerical curve rank (Huang et al. 2009) such that more potent and efficacious compounds with higher quality curves were assigned a higher rank, and inactive (class 4) compounds were assigned a curve rank of 0. Curve ranks should be viewed as a numerical measure of compound activity. Compounds with curve ranks > 4 or < –4 were considered as active activators or inhibitors, and compounds with other non-zero curve ranks were considered inconclusive.

The percentage of actives varied from as little as 0.07% for an epigenetics cell-based assay [Locus DeRepression (LDR)] (Figure 1) to as much as 41% for a biochemical assay that evaluated the ability of compounds to interact with cytochrome P450 CYP1A2 (Figure 3). Within any single assay, the potency of the active compounds (based on AC_50_ values) varied as much as five orders of magnitude.

Although the focus of qHTS at the NCGC is on screening large numbers of compounds in biochemical and cell-based assays of potential toxicological interest, the platform can also be used to explore genetic differences in sensitivity to toxicants. During Phase I, we evaluated differential sensitivity among a genetically defined panel of 81 human lymphoblastoid cell lines [27 Centre d’Etude du Polymorphisme Humain (CEPH) trios (parents and offspring) assembled by the HapMap Consortium (http://hapmap.ncbi.nlm.nih.gov/)] using 240 compounds (12 concentrations, 0.26 nM to 46 μM), a selected subset of the NTP library. Caspase 3/7 activity, a marker of apoptosis, and intracellular ATP, a measure of cell viability, were the end points evaluated. qHTS screening in the genetically defined population produced robust results, allowing for cross-compound, -assay, and -individual comparisons (Lock et al. 2012). The generation of high-quality qHTS *in vitro* cytotoxicity data for these genetically defined cell lines on a large library of compounds demonstrated the potential of this methodology to assess the degree of interindividual variability in toxicity and to explore its genetic determinants.

Phase I screening provided a valuable experience in the use of qHTS approaches for the toxicity screening of environmental compounds. The promise of this approach was clear, but limitations were also identified. Significant limitations on assay protocols are imposed by the use of 1,536-well plates on a highly automated robotics platform [National Center for Advancing Translational Sciences (NCATS) 2013a]. Assay selection is often constrained by currently available technologies, but the NCGC has been able to adapt many assays to conform to the technological requirements of qHTS. Through Small Business Innovative Research and Small Business Technology Transfer grants and contracts (NIH 2013b, U.S. EPA 2013c), research collaborations, and communications with commercial assay suppliers, the development of *in vitro* assays compatible with qHTS requirements has increased. Furthermore, to advance the capabilities of Tox21, there is a public nomination process for assays to be considered for implementation in qHTS (NCATS 2013c; NTP 2013d; U.S. EPA 2013d).

The results of the first qHTS cytotoxicity and apoptosis assays suggested the need to decide on a preferred origin of cells for cell-based assays. A goal of Tox21 is to use human cell–based as opposed to rodent cell–based assays whenever possible to eliminate concerns about species-dependent differences in response. In addition to considering species and tissue of origin, the use of primary cells or mixed cell cultures versus established, commercially available individual cell lines was explored. From a biological perspective, primary cells and mixed cell cultures would be preferred, but they present challenges of availability, generally require special handling, are not easily adaptable to 1,536-well assay conditions, and are not used to establish reporter gene assays, which constitute the majority of current qHTS assays. An issue that has been extensively discussed is whether to restrict assays, including gene transactivation assays, to a single cell type to reduce the number of variables affecting data interpretation or, alternatively, to select each assay based solely on maximizing sensitivity and reproducibility. Because of the limited availability of reporter gene assays using a common cell type, Phase II will employ the latter approach.

There are other technical limitations in the current qHTS paradigm. There is currently no method for including metabolic activation in the qHTS screens because liver S9 mix is toxic to cells when used beyond a few hours and the current qHTS assay protocols cannot include aspiration steps. Thus, there is a critical need to develop other approaches for including xenobiotic metabolism. These may include culturing primary hepatocytes alone or with a co-cultured reporter gene assay, culturing three-dimensional liver model inserts (which are currently not applicable to high throughput) into wells along with a co-cultured reporter gene assay, or using metabolically competent cell lines [e.g., HepaRG (Kanebratt and Andersson 2008)] as the target cell population. There are also targets of toxicological importance, such as the proteins involved in gap junction cell-to-cell communication or the orphan nuclear receptor constitutive androstane receptor, for which there are no existing *in vitro* assays amenable to qHTS.

The findings generated during Tox21 Phase I have demonstrated the applicability of the qHTS approach for screening a large library of environmental compounds. Assays originally developed for drug discovery can be used, directly or with modification, to evaluate cellular processes potentially involved in toxicity responses. Statistical approaches have been developed to analyze the enormous amounts of data produced from qHTS screens. However, data analysis has not been straightforward. A surprising number of complications have been identified and approaches to deal with these complex issues are discussed below.

Taking NCGC assay throughput into account, the experience in Phase I, and the results of a comprehensive analysis of disease-associated cellular pathways (e.g., Gohlke et al. 2009), the Phase II assay strategy is to initially focus on assays that measure the induction of stress response pathways (Simmons et al. 2009) and interactions with nuclear receptors. The selection of stress response assays (e.g., apoptosis, antioxidant response, cytotoxicity, DNA damage response, endoplasmic reticulum stress response, heat shock, inflammatory response, mitochondrial damage) is based on the premise that compounds that induce one or more stress response pathway are more likely to exhibit *in vivo* toxicity than those that do not. The human nuclear receptor assays (androgen; aryl hydrocarbon; estrogen-α; farnesoid X; glucocorticoid; liver X; peroxisome proliferator-α, -δ, and -γ; progesterone; pregnane X; retinoid X; thyroid-β; vitamin D) were selected because of the key roles they play in endocrine and metabolism pathways. The initial nuclear receptors assayed during Phase I at the NCGC used partial receptors that consisted of the ligand-binding domain and the C-terminal end (Huang et al. 2011a). However, because of concerns about potential differences in chemical response profiles when using a complete versus a partial receptor, the Phase II 10K library is being screened against both partial and complete receptors, at least for the androgen and estrogen receptors, in agonist and antagonist modes.

The primary limitation of this qHTS platform is that, although thousands of compounds can be screened in a single assay, each assay is generally limited, in terms of biological output, to one or two signals. In addition, most transcriptional activation assays are developed in established cell lines (and not always ones of human origin) rather than “normal” human cells. To potentially overcome these limitations, Tox21 is investigating several different genomic-based platforms that would allow for the monitoring of gene expression patterns (signatures), induction or repression, (e.g., 200–1,000) in any cell type, including human primary and stem cells (undifferentiated and differentiated) in a 384-well–plate format.

Also, in Tox21 Phase II, on the basis of the qHTS cytotoxicity results obtained with the 81 human lymphoblastoid cell lines (Lock et al. 2012), we (along with I. Rusyn and colleagues at the University of North Carolina–Chapel Hill) expanded the scope of this interindividual differential sensitivity project to evaluate approximately 1,100 different human lymphoblastoid cell lines, with densely sequenced genomes representing nine races of humankind, to 180 toxicants, using the CellTiter Glo® cell viability assay (Promega) to assess cytotoxicity. The large number of human cell lines used allows for an analysis of genetic determinants associated with differential cytotoxicity *in vitro*.

*Informatics and lessons learned*. In Phase I, an extensive set of concentration–response data was generated on approximately 1,400 (only NTP compounds tested) or approximately 2,800 compounds (both NTP and U.S. EPA compounds tested) screened at the NCGC in approximately 70 qHTS assays (see Table 1 for which sets of compounds were screened in which assays). In addition, in ToxCast™ Phase I, the U.S. EPA obtained concentration–response curves on 309 unique compounds tested across approximately 550 *in vitro* and lower organism *in vivo* assays by various contract and government laboratories (Chandler et al. 2011; Dix et al. 2007; Houck et al. 2009; Judson et al. 2010; Knight et al. 2009; Knudsen et al. 2011; Martin et al. 2009a, 2009b, 2010, 2011; Padilla et al. 2012; Rotroff et al. 2010). The raw data from HTS studies is generated using a number of different readouts (e.g., fluorescence, luminescence, phenotypic). Regardless of the assay readout, the goal is the same—identification of compounds that are active, not active, or inconclusive (i.e., based on the response, the compound is not clearly active or clearly inactive). In qHTS at the NCGC, the raw data generated at each chemical concentration tested are first normalized relative to the positive control response (i.e., 100% response) and the basal response in the solvent control DMSO-only wells (i.e., 0%) on the same 1,536-well plate, and then corrected by applying a pattern correction algorithm using the compound-free DMSO control plates. Outlier values are identified and removed based on the fit to the Hill equation, which is often used to describe sigmoidal biochemical phenomena [for a more extensive description of this process, see Inglese et al. (2006) and Shockley (2012)]. Traditional methods used to assess the significance of nonlinear regression analyses rely heavily on human inspection of individual residual plots or comparisons of the fit and the raw data, which is not practical in the qHTS analysis context with thousands of compounds in hundreds of assays. Furthermore, confounding effects such as autofluorescence of cellular constituents or the chemicals under study and cytotoxicity may complicate the data analysis and interpretation. The normalized and processed data set emerging from qHTS studies at the NCGC is very large, and a number of heuristic approaches and statistical models have been developed to address a variety of HTS data structures (Inglese et al. 2006; Parham et al. 2009; Shockley 2012).

When concentration–response data can be confidently modeled, AC_50_ values are calculated from curve fits to the three- or four-parameter Hill equation. In assay sets in which no upper asymptote (agonist assays) or lower asymptote (antagonist or cytotoxicity) can be defined, a lowest effective concentration is calculated, defined as the lowest concentration at which there is a statistically significant difference from the concurrent negative control. The results are corrected to remove artifacts due to cytotoxicity and parameters such as concentration for half maximal activity, maximum efficacy, and minimal response are used to make activity calls based on algorithms for specific assay types and platforms (Judson et al. 2010).

The extensive data being generated by Tox21, both in terms of the number of compounds being screened and in the diversity of assays being used, is providing a unique opportunity for the development of novel approaches for making activity calls and for relating those calls to “truth” based on existing human and animal data. The determination of which approach is most appropriate depends on *a priori* knowledge of the assay in question, the purpose of the study, and the structure of the data. The collection of Tox21 data will be used to create a diagram of the biological network that responds to chemical perturbations that will be linked to toxicological effects in animals and humans. To achieve this goal, assays that measure targets that encompass pathways relevant to toxicity need to be used. However, there is no single comprehensive and uniform resource that covers all known annotations of pathways or any single platform that allows integrated browsing, retrieval, and analysis of information from the many existing individual web-based pathway resources. In response to this need, the NCGC is building an integrated pathway resource that hosts information from manually curated and publicly available resources. The NCGC Human BioPlanet will allow easy browsing, visualization, and analysis of the universe of human pathways. The main view of the BioPlanet maps all known human pathways on a three-dimensional globe, where each spot represents a gene on a pathway. Selecting a gene on the globe will place all elements of the pathway in a detailed view window. Detailed descriptions of all genes in the selected pathway are shown below the three-dimensional graphics. When multiple pathways are selected at the same time, the view will show all unique gene components within selected pathways. Multiple pathways can be illustrated as one extended pathway that better shows the interaction between different biological processes. The BioPlanet will be searchable by any gene or pathway identifier and also by disease relevance (prevalence of disease genes), toxicity relevance (occurrence of genes in toxicology literature), and availability of assays in PubChem. Thus, this platform will allow for an assessment of assay coverage across the approximately 1,100 human pathways and where new assays might be most useful.

The qHTS assays used at the NCGC focus on cell-based phenotypic or transactivation end points and do not measure directly the binding of a compound to a receptor or other cellular component. However, the wealth of data being generated in Tox21 Phase II will be used to both test the validity of existing docking and quantitative structure activity relationship (QSAR) models and for developing new ones. Given the public availability of the structures of the 8,193 unique compounds included in the Phase II > 10K library (U.S. EPA 2013e), we invite the scientific informatics community to predict the activity of these compounds in the different nuclear receptor and stress response pathway assays before public release of the data.

One critically important goal is to make all Tox21 data publicly accessible via various databases, including the NTP’s Chemical Effects in Biological Systems (CEBS; NTP 2013a), the U.S. EPA’s Aggregated Computational Toxicology Resource (ACToR; Judson et al. 2012, U.S. EPA 2013a), PubChem, and the NCATS Tox21 Chemical Browser (NCATS 2013b), to encourage independent evaluations of Tox21 findings. Data will be made available after ensuring accurate linkage of the compound to its correct structure, the results of the chemical analysis, and assay responses.

*Will it work?* Although we’ve made good progress in laying the groundwork to enable us to answer the question of whether Tox21 can fulfill the expectations to transform toxicity testing, the area that requires the most work is one that we term targeted testing. This term encompasses everything from designing and carrying out confirmatory assays for a given biological outcome in a second related *in vitro* assay (i.e., an orthogonal assay); incorporating engineered human tissue models into Tox21; and confirming a response in a whole organism such as *Caenorhabditis elegans*, zebrafish, or rodents to evaluating methods for extrapolating from *in vitro* concentration to *in vivo* dose levels. Perhaps the most important type of targeted testing that must be accomplished is the simple but huge intellectual effort needed to compare the output of Tox21 with what we know from our existing databases of animal and human toxicology. To date, data from ToxCast™ have been used to develop a number of prediction models and prioritization schemes (Judson et al. 2008, 2010, 2011; Kleinstreuer et al. 2011a, 2011b, 2013; Martin et al. 2009a, 2009b, 2011; Reif et al. 2010; Rotroff et al. 2013; Sipes et al. 2011). qHTS data generated at the NCGC have been used to develop structural feature models (Huang et al. 2009), to profile the chemical modulation of multiple human nuclear receptors (Huang et al. 2011a), to evaluate chemicals capable of interfering with mitochondrial function (Sakamuru et al. 2012), and to predict chemical structures that interact with cytochrome P450 (Sun et al. 2012) among others. Although predictive models of phenotypic outcomes will require considerable effort to evaluate their reliability and relevance to support regulatory action, the technologies employed in the Tox21 program are actively being investigated for application to the prioritization of chemicals in testing programs by the U.S. EPA. For example, in the EDSP21 program (U.S. EPA 2011, 2013b), the short-term goal is to use the technologies to prioritize chemicals for nomination for screening in the current EDSP assay battery, whereas the intermediate- and long-term goals target the incorporation and ultimate replacement of the current assays with *in silico* and molecular-based high throughput assays.

In support of Tox21, the NTP is evaluating techniques for mining its tissue archives for gene expression response profiles to expand our ability to link chemicals to genes, genes to pathways, and pathways to disease. The NTP archives contain stained histopathology slides, paraffin tissue blocks, formalin-fixed tissues and organs, and selected frozen tissue from over 2,000 experimental rodent studies, including toxicity, carcinogenicity, immunotoxicity, reproductive, and developmental studies. We have conducted studies to evaluate the extent to which gene expression signatures can be reliably derived from the molecular analysis of tissue samples stored as formalin-fixed, paraffin-embedded tissues (Merrick et al. 2012). Such signatures could contribute to a more comprehensive understanding of dose–response relationships at the molecular level, the identification of useful targets for *in vitro* assays, to an evaluation of the correlation between *in vitro* test results and *in vivo* toxicological outcomes, and to the development of predictive models of toxicity.

In addition, the NTP recently acquired DrugMatrix®, a toxicogenomics reference database, tissue archive, and informatics system originally developed by Iconix (Mountain View, CA) in 2007. The NTP acquired this resource in order to expand our ability to develop predictive models for toxicological effects based on gene signatures, to provide an additional tool for linking *in vitro* data to *in vivo* gene signatures and disease outcomes, and to provide additional tissue samples for next generation sequencing–based investigations. This integrated database of rat gene expression profiles, pathology measures, pharmacology assays, and literature information on 657 compounds, primarily drugs along with the linked automated toxicogenomics analysis application, ToxFX® are publicly accessible (NTP 2013b, 2013c). ToxFX® is useful for formulating gene signatures of toxicity, for identifying potentially useful targets for *in vitro* assays, for linking *in vitro* data to *in vivo* toxicological effects, and for evaluating the extent to which humans and rodents share common toxicity/disease pathways. The U.S. EPA’s NCCT, in its virtual embryo (U.S. EPA 2013g) and virtual liver (U.S. EPA 2013h) projects, are building a knowledgebase of chemical effect networks to produce computable models of key molecular, cellular, and circulatory systems in the human liver and the developing embryo, respectively.

## Conclusion

We fully appreciate that Tox21 faces some very difficult issues:

“Perfect” assays do not exist.
Coverage of all chemicals of interest is incomplete (i.e., volatiles).
A high throughput system for measuring the free concentration of a compound *in vitro* is not yet available.
Xenobiotic metabolism is lacking in virtually all *in vitro* assays.
Interactions between cells are poorly captured.
Distinguishing between statistical and biological significance is difficult.
Extrapolating from *in vitro* concentration to *in vivo* dose or blood levels is not straightforward.
Assessing the effects of chronic exposure conditions *in vitro* is not possible.
Identifying when a perturbation to a gene or pathway would lead to an adverse effect in animals or humans remains a challenge.
Achieving routine regulatory acceptance of the developed prediction models is years away.

However, we are making progress in integrating data from diverse technologies and end points into what is effectively a systems biology approach to toxicology. This can only be accomplished when comprehensive knowledge is obtained with broad coverage of chemical and biological/toxicological space. The efforts described thus far reflect the initial stage of an exceedingly complicated program, one that will likely take decades to fully achieve its goals. However, even at this stage, the information obtained is enticing the international scientific community and, we believe, foretelling the future of toxicology.
